# Comparative Molecular Field Analysis of Benzothiazepine Derivatives: Mitochondrial Sodium Calcium Exchange Inhibitors as Antidiabetic Agents

**DOI:** 10.4103/0250-474X.41453

**Published:** 2008

**Authors:** A. S. Dasoondi, V. Singh, S. R. Voleti, Meena Tiwari

**Affiliations:** 1 Computer Aided Drug Design Group, Department of Pharmacy, S. G. S. I. T. S, 23-Park Road, Indore-452 003, India; 2 Molecular Modeling Laboratory, NDDR, Ranbaxy Research Laboratories, Plot No. 20, Sector-18, Udyog Vihar Industrial Area, Gurgaon-122 001, India

**Keywords:** CoMFA, 3D-QSAR, antidiabetic agents, mitochondrial sodium calcium exchange, benzothiazepines

## Abstract

Mitochondrial sodium calcium exchange inhibitors are novel agents in the treatment of type-II diabetes due to their glucose dependent efficacy. While the compounds of this class are expected to correct hyperglycemia, they do not lower basal blood glucose level, thus avoiding the serious consequences of hypoglycemia. The 3DQSAR analysis of benzothiazepines and their derivatives as mitochondrial sodium calcium exchange inhibitors was performed by comparative molecular field analysis to determine the structural factors required for the activity of these compounds. After performing a leave one out cross validation study, satisfactory results were obtained, with cross-validated q^2^ and conventional r^2^ values of 0.711 and 0.970, respectively. The results provided the tools for predicting the affinity of the related compounds, and guidance for the designing and synthesis of novel and potent mitochondrial sodium calcium exchange inhibitors as antidiabetic agents.

Diabetes mellitus, Type-II, is a chronic metabolic disorder, accounting for highest number of diagnosed diabetes cases. Impaired insulin secretion, insulin resistance and excessive hepatic gluconeogenesis, affecting protein and lipid metabolism leading to serious cardiovascular, renal, neurological and retinal complication, characterize it^1^–^2^. The incidence of such complication can be reduced if the blood glucose level is maintained within normal range. The current therapy includes insulins, insulin secretogogues (sulphonylureas and metiglinides), insulin sensitizers (biguanides and thiazolidinediones), inhibitors of intermediary metabolism (antihyperlipidemic drugs), inhibitor of glucose uptake (acarbose, pramlinitide), and insulinomimetic drugs. But their mechanism related side effects (weight gain, hypoglycemia, gastric intestinal distress) limits their efficacy for prolonged use. The commonly used sulfonylureas may lose their efficacy after prolonged drug treatment as a result of over stimulation of pancreatic β-cells, which leads to β-cells fatigue. Not only this, insulin secretogogues available also stimulate insulin secretion under fasting condition leading to serious consequences of hypoglycemia^3^–^7^.

Recently, mitochondrial sodium calcium exchanger (mNCE) has been investigated as a novel target for diabetes drug discovery. It has been demonstrated that inhibition of mNCE increases the magnitude and duration of glucose induced transient rise in mitochondrial Ca^2+^ concentration and results in glucose stimulated insulin secretion in the β-cells. The advantage of these agents is their glucose dependent efficacy against hyperglycemia with no lowering of fasting/basal blood glucose level, thus avoiding the liability of hypoglycemia^8^–^12^. Compounds with different basic structures such as 1,4-benzothiazepine-2-one (CGP3757), 1,5-benzothiazepine-2-one (diltiazem), 1,4-benzdiazepine-2-one (clonazepam) showed mNCE inhibitory activity. 1,4-benzothiazpine-2-one is the most potent inhibitor having IC50 value of 0.4 μM but its low solubility and short half-life limits its use for preclinical studies. Only few numbers of candidates as NCE inhibitors and a little information about the structure activity relationship, greatly affect the pharmacological studies of these agents^13^.

Through this paper, we describe 3D-QSAR/CoMFA studies of the Benzothiazepines and their derivatives, obtained from literature. The model obtained could be effectively utilized as a guiding tool for further structure modification and synthesis of new potent mNCE inhibitors as antidiabetic agents.

## Materials and Methods

### Data set for manipulation:

**Fig. 1:**
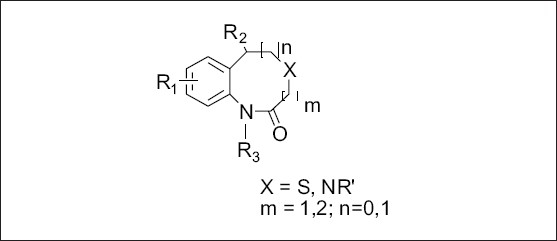
General structure of thiazolidinone derivatives.

A diverse set of 36 Benzothiazepines and their derivatives was taken from the literature^14^. The structure of the compounds used in the study and their biological activity IC50 values μM (inhibition of mNCE mediated Na^+^/Ca^2+^ translocation in mitochondria in permeabilized cells monitored, using Ca^2+^ sensing fluorescence, in the presence of drug), expressed as pIC50 (-logIC50) are given in Tables 1 and 2. The general structure of Benzothiazepines and their derivatives is shown in fig. 1. The pIC50 was used as dependent variable in the QSAR study. The whole data set was randomly divided into two subsets, the training set and test set containing 29 and 7 data points, respectively. The training set of Benzothiazepines and their derivatives was used for 3D-QSAR analysis. In addition, 7 compounds selected with a good variation in the basic structure of Benzothiazepines, were kept to test the actual prediction of the model.

**TABLE 1:** TRAINING SET MOLECULES AND THEIR MNCE INHIBITORY ACTIVITY

No.	R1/R.	R2	R3	X	IC_50_* (μM)	pIC_50_# (M)
1	Cl	3-Me-C_6_H_4_-	H	S	12.6	4.9
2	Cl	4-Me-C_6_H_4_-	H	S	39.8	4.4
3	Cl	2,3-diMe-C_6_H_3_-	H	S	10.0	5.0
4	Cl	2,5-diMe-C_6_H_3_-	H	S	25.1	4.6
5	Cl	2,6- diMe-C_6_H_3_-	H	S	25.1	4.6
6	Cl	3,4- diMe-C_6_H_3_-	H	S	20.0	4.7
7	Cl	3,5- diMe-C_6_H_3_-	H	S	15.9	4.8
8	Cl	2-benzthiazolyl	H	S	20.0	4.7
9	Cl	2-thiophenyl	H	S	25.1	4.6
10	NO_2_	C_6_H_5_-	H	S	20.0	4.7
11	H	2-Cl-C_6_H_4_-	H	S	15.9	4.8
12	H	2-Me-C_6_H_4_-	H	S	25.1	4.6
13	Cl	Cyclohexyl	H	S	15.9	4.8
14	Cl	Isopropyl	H	S	50.1	4.3
15	Cl	Isobutyl	H	S	25.1	4.6
16	Cl	2-Cl-C_6_H_4_-	N(Me_2_)CH_2_CH_2_	S	39.8	4.4
17	Cl	2-Cl-C_6_H_4_-	Acetyl	S	20.0	4.7
18	Cl	2-Cl-C_6_H_4_-	H	EtOCH_2_CH_2_CH_2_N-	3.2	5.5
19	Cl	2-Cl-C_6_H_4_-	H	HO CH_2_CH_2_-	7.9	5.1
20	Cl	2-Cl-C_6_H_4_-	H	2-(Pr)_2_N CH_2_CH_2_N-	6.3	5.2
21	Cl	2-Cl-C_6_H_4_-	H	(MeO CH_2_CH_2_)_2_NC CH_2_CH_2_N-	2.0	5.7
22	Cl	2-Cl-C_6_H_4_-	H	3,4-(MeO)_2_PhCH_2_CH_2_N-	5.0	5.3
23	Cl	2-Cl-C_6_H_4_-	H	S	2	0
24	Cl	2-Cl-C_6_H_4_-	H	S	1	1
25	Cl	2-F-C_6_H_4_-	H	S	1	1
26	C_6_H_5_-	-	-	N	159.0	3.8
27	4-Me-C_6_H_5_-	-	-	N	100.0	4.0
28	C_6_H_5_-	-	-	NH	63.1	4.2
29	4-Me-C_6_H_5_-	-	-	NH	79.4	4.1

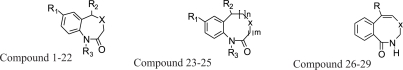						

* IC_50_(μM) = Inhibition of mNCE mediated Na^+^/Ca^2+^ translocation in mitochondria in permeabilized cells monitored, using Ca^2+^ sensing fluorescence, in the presence of drug # pIC_50_ (M)= -logIC_50_(M)

**TABLE 2:** TEST SET MOLECULES AND THEIR MNCE INHIBITORY ACTIVITY 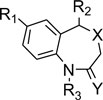

No.	R_1_	R_2_	R_3_	X	Y	IC_50_* (μM)	pIC_50_# (M)
30	Cl	2-Cl-C_6_H_4_-	H	S	O	1.40	5.85
31	Cl	C_6_H_5_-	H	S	O	12.60	4.90
32	Cl	2-Me-C_6_H_4_-	H	S	O	6.30	5.20
33	Cl	2-thiazolyl	H	S	O	200	3.70
34	Cl	4-pyridyl	H	S	O	31.60	4.50
35	Cl	3-BnO-Pr-	H	S	O	3.20	5.50
36	Cl	2-Cl-C_6_H_4_-	H	S	H,H	6.30	5.20

* IC_50_(μM) = Inhibition of mNCE mediated Na^+^/Ca^2+^ translocation in mitochondria in permeabilized cells monitored, using Ca^2+^ sensing fluorescence, in the presence of drug # pIC_50_ (M)= -logIC_50_(M)

### Molecular modeling:

**Fig. 2:**
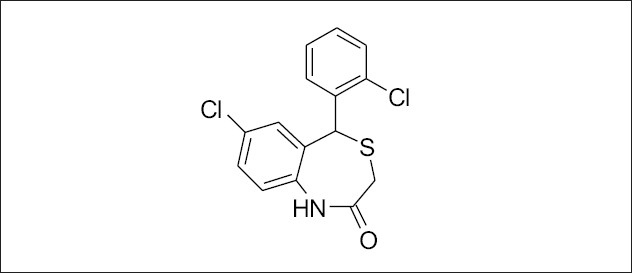
Template for alignment of molecules.

All molecular modeling techniques and 3D QSAR studies described herein were performed on SGI/IRIX 6.5 workstation using SYBYL 6.9.1 molecular modeling software^15^. Since the structural information on these inhibitor protein complexes is not available, therefore, the use of low energy conformation in the alignment is a useful starting point for statistical comparison of flexible structure within the CoMFA models. In this study, atom based alignment methods were used which involves atom based fitting (RMS fitting) of the ligands. The compounds were fitted to the template molecule as shown in (fig. 2). The energy minimization of all the compounds was performed using molecular mechanics with the MMFF94 force field with a 0.05 kcal/mole energy gradient convergence criterion. Charges were calculated by the MMFF94 method at the beginning and Gasteiger-Hükel charges were considered for further calculations.

### Molecule alignment:

The most important requirement for CoMFA studies is that the 3D structure of the molecules to be analysed be aligned according to suitable conformational template, as the resulting 3D QSAR model is often sensitive to the particular alignment scheme^15^. The selected template molecule is the most active compound or the lead and/or commercial compound or the compound containing the greatest number of functional groups or the low energy conformation of the most active compound is set as reference^16^–^18^. The conformational search was performed using the multisearch routine in SYBYL. Compound 7 (most active compound) was chosen as the template molecule, on which other molecules were aligned.

**Fig. 3:**
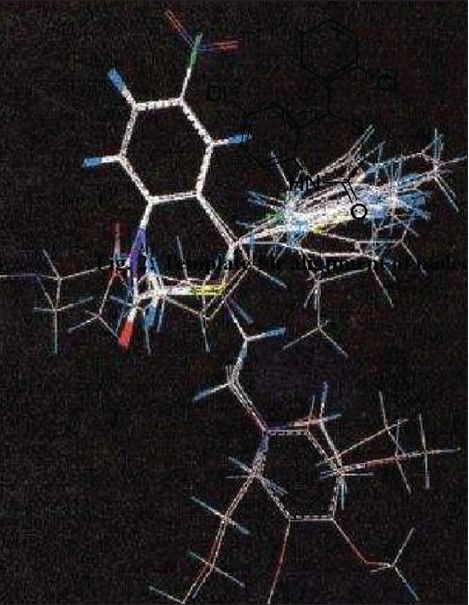
3D-views of all aligned compounds by rms fitting. The atom fit molecular alignment method (RMS fitting) was adopted by minimizing the rms distance between atoms pairs, respectively, to the fitting molecule and to the template molecule.

In the present study, the atom fit molecular alignment method^19^–^20^ (RMS fitting) was adopted by minimizing the rms distance between atoms pairs belonging, respectively, to the fitting molecule and to the template molecule as shown in fig. 3.

### Comparative molecular field analysis:

Following alignment, the molecules were placed one by one into the 3D cubic lattice with 2Ågrid. The steric (Legnard-Jones 6-12 potential) and electrostatic (Coulomb potential) fields were calculated with a distance dependent dielectric at each grid point using a sp^3^ hybridized carbon probe with a +1.0 charge. A 30-kcal/mol energy cutoff was applied, which means that the steric and electrostatic energies greater than 30kcal/mol was truncated to that value, thus, can avoid infinite energy values inside molecules. The CoMFA steric and electrostatic fields generated were scaled by the CoMFA-STD method in SYBYL.

To derive 3D-QSAR models, partial least square analysis, using the standard SYBYL implementation, was used. The biological activities (pIC50) were correlated with the CoMFA values that contain the magnitude of either the steric or electrostatic potentials. To avoid over fitted 3D-QSAR, the optimum number of components (N) were chosen which gave less standard error of prediction and highest cross-validated correlation coefficient (q^2^ ). The cross validated q^2^ was calculated using following Eqn., q^2^ = 1- (∑ (Y-Y_pred_)^2^)/∑(Y-Y_mean_)^2^

The cross-validated q^2^ quantifies the predictive ability of the model. It was determined by a leave-one-out (LOO) method of cross-validation in which each compound is successively removed from the model derivation and its pIC_50_ value predicted using the model built from the remaining compounds. A “region focusing” was applied using the deviation coefficients values as weights, to the lattice points in a CoMFA region to enhance or attenuate the contribution of lattice point in subsequent analysis. The experimental factors in the “Sigma field” default option were kept low to 0.02 to 0.05 to control the sharpness of focusing. To speed up the analysis and reduce noise, column filtering was set at 0.2 kcal/mol so that only those steric and electrostatic energies having values greater than 2.0 kcal/mol were considered in the PLS analysis. Using these stages, the predictive quality of the best model was determined.

A progressive scrambling based test was performed to determine the sensitivity of QSAR model to chance correlation. The slope of q^2^ evaluated at the specific critical point with respect to the correlation of the original dependent variables versus the perturbed dependent variables (dq^2^/dr^2^) was 0.977, suggested that the model was stable and do not change greatly with small changes in underlying data set. QSAR models which are unstable changes greatly with small changes in underlying data set characterized by slope greater than 1.20. Stable models have slope near unity. At the same time, the CoMFA color contour maps were derived for the steric and electrostatic fields. The statistical parameters q^2^, which indicate the good predictability of the model if having value greater than 0.40 and r^2^, which shows the self consistency of the model if having value greater 0.90, are measures of the quality of the final CoMFA model.

## RESULTS AND DISCUSSION

**Fig. 4:**
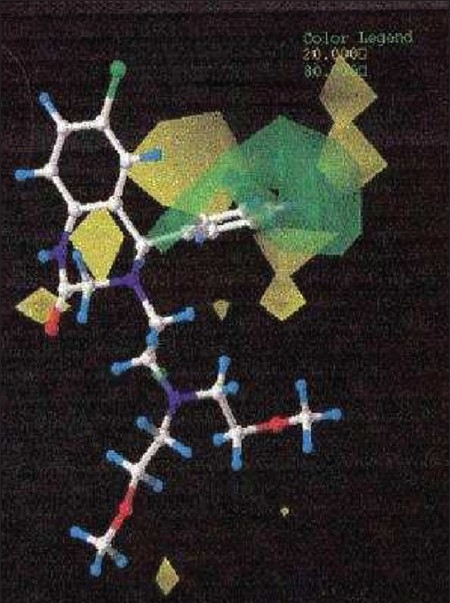
The Steric contour maps of CoMFA model. The green isopleths shows favorable green areas with more bulk (std. dev × coeff.), where as yellow isopleths shows unfavorable steric areas (std. dev × coeff.).

**Fig. 5:**
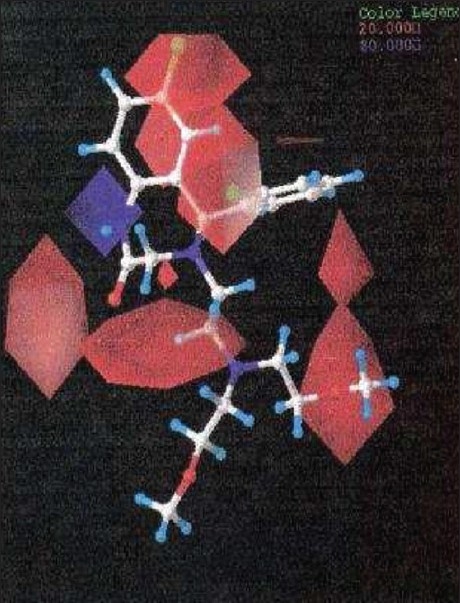
The Electrostatic contour maps of CoMFA model. The favorable electrostatic areas (std. dev × coeff.) with positive charges are indicated by blue isopleths, whereas the favorable electrostatic areas (std. dev × coeff.) with negative charges are indicated by red isopleths.

The results from the CoMFA studies are summarized in Table 3. The rms fitting alignment with Gasteiger-Hukel charges showed a cross validated q^2^= 0.711 with five components. A non cross-validated r^2^= 0.970 with F value= 150.933 was also observed. In the analysis, almost equal contribution was observed from steric (52.7%) and electrostatic (47.3%) fields. The experimental and predicted IC_50_ values for the training set having very small difference (maximum error = 0.18) are summarized in Table 4. Graph of the actual versus predicted pIC_50_ values for the training set is shown in fig. 4. Also, generated model was used to predict the activity of the test set molecules summarized in Table 5 and graph plotted between the actual and predicted activity of test set molecules is shown in fig. 5.

**TABLE 3:** SUMMARY OF CoMFA MODEL

PLS statistics	Value
Cross validated correlation coefficient, q^2^	0.711
Non cross validated correlation coefficient, r^2^	0.970
Number of components (N)	5
Standard error of estimation (ESS)	0.081
F-value	150.933
Steric	52.7%
Electrostatic	47.3%

*The q^2^ indicates good predictability of the model (value greater than 0.40), r^2^ shows the self consistency of the model having value greater 0.90

**TABLE 4:** EXPERIMENTAL AND PREDICTED ACTIVITIES OF THE TRAINING SET MOLECULES

No.	Experimental (pIC_50_*, M)	Predicted (pIC_50_, M)	Error
1	4.90	4.86	0.04
2	4.40	4.51	-0.11
3	5.00	4.99	0.01
4	4.60	4.67	-0.07
5	4.60	4.65	-0.05
6	4.70	4.74	-0.04
7	4.80	4.92	-0.12
8	4.70	4.70	0.00
9	4.60	4.63	-0.03
10	4.70	4.58	0.12
11	4.80	4.74	0.06
12	4.60	4.70	-0.10
13	4.80	4.62	0.18
14	4.30	4.38	-0.08
15	4.60	4.58	0.02
16	4.40	4.29	0.11
17	4.70	4.71	-0.01
18	5.50	5.54	-0.04
19	5.10	5.10	0.00
20	5.20	5.16	0.04
21	5.70	5.71	-0.01
22	5.30	5.29	0.01
23	4.90	4.92	-0.02
24	5.20	5.10	0.10
25	4.80	4.79	0.01
26	3.80	3.91	-0.11
27	4.00	3.95	0.05
28	4.20	4.13	0.07
29	4.10	4.12	-0.02

* pIC_50_ (M)= -logIC_50_(M). Experimental values are the biological activity values (IC50, in μM, inhibition of mNCE mediated Na^+^/Ca^2+^ translocation in mitochondria in permeabilized cells monitored, using Ca^2+^ sensing fluorescence, in the presence of drug) of the molecules in training set, as reported in literature and Predicted values are CoMFA model values

**TABLE 5:** EXPERIMENTAL AND PREDICTED ACTIVITIES OF THE TEST SET MOLECULES

No.	Experimental (pIC_50_*, M)	Predicted (pIC_50_, M)	Error
1	5.85	4.76	1.09
2	4.90	4.62	0.28
3	5.20	4.74	0.46
4	3.70	4.66	-0.96
5	4.50	5.42	-0.92
6	5.50	4.79	0.71
7	5.20	4.75	0.45

* pIC_50_ (M)= -logIC_50_(M). Experimental values are the biological activity values (IC50, in μM, inhibition of mNCE mediated Na^+^/Ca^2+^ translocation in mitochondria in permeabilized cells monitored, using Ca^2+^ sensing fluorescence, in the presence of drug) of the molecules in test set, as reported in literature and Predicted values are CoMFA model values

**Fig. 6:**
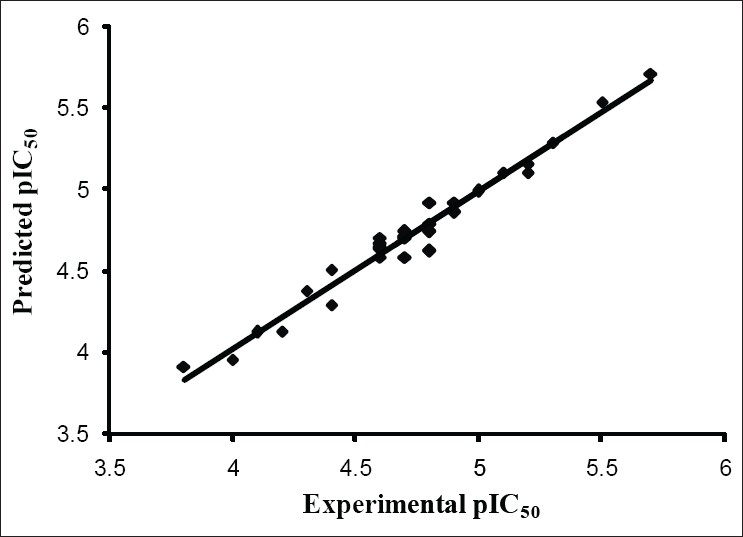
Predicted vs. experimental pIC_50_ values of the training set. Experimental values are the biological activity values (IC_50_, in μM, inhibition of mNCE mediated Na^+^/Ca^2+^ translocation in mitochondria in permeabilized cells monitored, using Ca^2+^ sensing fluorescence, in the presence of drug) of the molecules in training set, as reported in literature and Predicted values are CoMFA model values, r^2^ 0.970.

**Fig. 7:**
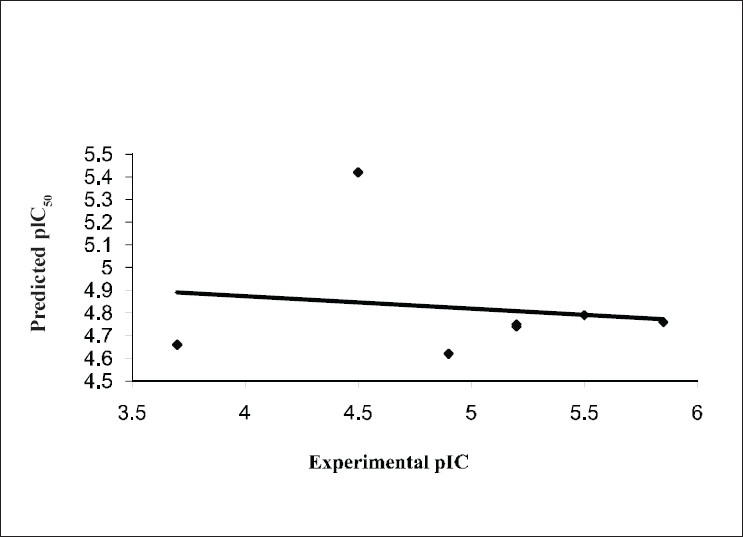
Predicted vs. experimental pIC50 values of the test set. Experimental values are the biological activity values (IC_50_, in μM, inhibition of mNCE mediated Na^+^/Ca^2+^ translocation in mitochondria in permeabilized cells monitored, using Ca^2+^ sensing fluorescence, in the presence of drug) of the molecules in test set, as reported in literature and Predicted values are CoMFA model values, q^2^ 0.711.

The CoMFA steric and electrostatic fields based PLS analysis are represented as 3D contour plots in figs. 6 and 7, using compound 18 as reference structure. The steric contour map shows that the phenyl ring attached at C-5 of the molecules is surrounded by sterically favored region that are flanked with a small unfavorable yellow region, suggesting that there is a definite requirement of substructure with appropriate shape to exhibit biological activity at C-5 of the molecule. This is further supported by analyzing compounds 9, 33, 14 and 15. Compound 9 and 33, have a penta ring attached at C-5 but greatly differ in their activity (Compound 9 has pIC_50_ = 4.60 and compound 33 has pIC_50_ = 3.7) because attachment is having different orientation. Compound 14 and 15 both have aliphatic chain system at C-5 but compound 15 is more active (pIC_50_ = 4.6) than compound 14 (pIC_50_ = 4.3) because compound 14 has isopropyl group at C-5 and compound 15 has more bulky isobutyl at same place.

There are positive charge favorable blue contours found near nitrogen atom present at first position in the benzothiazepine skeleton suggesting that there is a requirement of positive charge atom (N) at this position. This is further supported by compounds 26, 27, 28, 29, all these compounds do not have N atom at first position in benzothiazepine skeleton and have low pIC_50_ values 3.80, 4.0, 4.2 and 4.1, respectively.

The contours also show negative charge favorable red polyhedral near C-7 of benzothiazepine skeleton and C’-2 of the phenyl ring attached at C-5 of the skeleton and also at the ‘O’ atom in the side chain, attached at position 4 of the benzothiazepinone skeleton and C’’-2 of the side chain. Red contours near these positions of the skeleton structure shows that high electron density at these positions may play a favorable role in the mNCE inhibitory activity. Electron rich atom at the C-7, C´-2 of the phenyl ring attached at C-5 of the skeleton equally contributes to the inhibitory activity. This is supported by compound 26, 27, 28 and 29, which show decreased biological activities due to the lack of electron rich atom at the said position. Further analysis of compounds 10, 11 and 12, which have similar activities, proves the importance of electron rich atom at above position. Compound 12 (pIC_50_ = 4.60) does not have electron rich atom at position C-7 but CH_3_ at C´-2 of phenyl ring attached at C-5 of the benzothiazepine skeleton. In compound 10, (pIC_50_ = 4.7) electron rich group (NO_2_ ) is present at position C-7 but no electron rich atom/group at C´-2 of phenyl ring. Compound 11(pIC_50_ = 4.80) do not have electron rich atom at position C-7 but Cl group at C´-2 of phenyl ring.

In this study, the CoMFA studies on a set of Benzothiazepines and their derivatives were aimed to derive structural requirement for mNCE antagonists. The CoMFA model gave a good statistical result and provides a significant correlation of steric and electrostatic parameter with the mNCE inhibitory activity. From contour map it can be concluded that the steric bulk substituents at para position on phenyl group attached at the C-5 position of the molecules may increase the activity. Also, more electropositive atom in the place of nitrogen at position 1 and more electronegative atom at C-7 position and in the side chain of molecules may increase the activity. The information obtained in this study provides the tool for predicting the affinity of benzothiazepines and its derivatives, and for guiding further structural modification and synthesize new potent mNCE inhibitors as antidiabetic agents.
